# Impact of Truenat on TB diagnosis in Nigeria

**DOI:** 10.5588/pha.24.0021

**Published:** 2024-09-01

**Authors:** R. Eneogu, J. Olabamiji, A. Ihesie, N. Nwokoye, K. Ochei, P. Nwadike, O. Salau, Z. Munguno, B. Odume, A. Agbaje, D. Nongo, O. Oyelaran, W. van Germert, L. Mupfumi, E. Emeka, C. Anyaike, E.N. Ossai

**Affiliations:** ^1^ HIV AIDS & TB Office, USAID Nigeria, Abuja;; ^2^ HIV-Nigeria, Lagos, Nigeria;; ^3^ KNCV Nigeria, Abuja, Nigeria;; ^4^ USAID Leap Project, Abuja, Nigeria;; ^5^ Stop TB Partnership, Geneva, Switzerland;; ^6^ Federal Ministry of Health, Abuja, Nigeria;; ^7^ Department of Community Medicine, College of Health Sciences, Ebonyi State University Abakaliki, Nigeria.

**Keywords:** Truenat, diagnosis, Africa, tuberculosis

## Abstract

**OBJECTIVE:**

To evaluate the impact of Truenat assays on the diagnosis of TB at peripheral facilities in Nigeria.

**METHODS:**

This was a retrospective cross-sectional study across 34 out of 38 Truenat-implementing facilities in Nigeria. These facilities offer DOTS services. Information on turnaround time (TAT) for TB diagnosis and time to commencement of treatment was obtained. Also, trends in TB case notifications at the facilities 9 months prior to and 9 months during Truenat implementation were compared.

**RESULTS:**

Of 2,335 entries, 70.1% (1,636) were used to determine TAT, while 45.8% (*n* = 1,070) were used for time to treatment initiation. The median time to diagnosis was <24 hours (IQR 0–1 days). The majority (57.9%) were diagnosed in <24 hours. The median time to treatment initiation was 1.5 days (IQR 0–3). In 9 months before the introduction of Truenat, 8% of 20,424 presumptives evaluated were positive for TB, while during the implementation, 9% of 37,087 presumptives were positive for TB.

**CONCLUSION:**

Truenat deployment led to an increase in TB and DR-TB case detection in peripheral facilities in Nigeria. It also decreased TAT and time to TB treatment initiation. These have positive implications in the fight against TB, and Truenat is relevant in finding missing TB cases in Nigeria.

## Abstract (fr)

**OBJECTIF:**

Évaluer l'impact des tests Truenat sur le dépistage de la TB dans les centres de santé périphériques du Nigeria.

**MÉTHODES:**

Il s'agit d'une recherche transversale rétrospective menée dans 34 des 38 établissements ayant adopté le test Truenat au Nigeria. Ces établissements fournissent des services DOTS. Les données concernant le délai de diagnostic de la TB (TAT, pour l’anglais « turnaround time ») et le délai de début du traitement ont été recueillies. De plus, une comparaison a été faite sur la tendance de la notification des cas de TB dans les établissements neuf mois avant et neuf mois pendant la mise en œuvre de Truenat.

**RÉSULTATS:**

Sur un total de 2 335 entrées, 70,1% (1 636) ont été utilisées pour déterminer le TAT, tandis que 45,8% (*n* = 1 070) ont été utilisées pour déterminer le délai de mise en route du traitement. Le délai médian avant le diagnostic était inférieur à 24 h (IQR 0–1 jour). La majorité des cas (57,9%) ont été diagnostiqués en moins de 24 h. Le délai médian d'initiation du traitement était de 1,5 jour (IQR 0–3). Sur une période de 9 mois précédant l'introduction de Truenat, 8% des 20 424 présomptions évaluées étaient positives à la TB, tandis que pendant la mise en œuvre, 9% des 37 087 présomptions étaient positives à la TB.

**CONCLUSION:**

L'implémentation du Truenat a favorisé une augmentation de la détection des cas de TB et de DR-TB dans les centres de santé périphériques du Nigeria. De plus, cela a contribué à réduire le TAT et le délai d'initiation du traitement de la TB. Ces résultats ont un impact positif dans la lutte contre la TB, et le Truenat s'avère être un outil précieux pour repérer les cas de TB manquants au Nigeria.

Nigeria is one of the 30 high TB burden countries and among the high multidrug-resistant TB (MDR-TB) burden countries of the world. Nigeria is currently ranked fifth among the 10 countries that collectively account for 75% of the global gap between the estimated number of newly diagnosed patients with TB and the number of newly diagnosed and reported.^1^ This gap has been attributed to the underdiagnosis and under-reporting of people diagnosed with TB.^1^ The WHO estimates that approximately one in three people who developed MDR-TB and rifampicin (RIF) resistant (RR-TB) were enrolled in treatment in the year 2021.^1^ Nigeria is also listed among the 10 countries that account for approximately 70% of the global gap between the estimated global incidence of MDR/RR-TB each year and the number of people enrolled in treatment in 2021.^1^

The Truenat™ (Molbio Diagnostics, Verna, India) testing system uses portable, battery-operated devices to rapidly detect *Mycobacterium tuberculosis* complex (MTBC) and RIF resistance. The system involves two main devices: the Trueprep^®^ AUTO v2 Universal Cartridge-based Sample Prep Device (Molbio Diagnostics) for the automated extraction and purification of DNA and the Truelab^®^ Real Time Micro-PCR Analyzer (Molbio Diagnostics) for performing real-time polymerase chain reaction (PCR), resulting in the semi-quantitative detection of MTBC. The Truenat system is primarily designed to be operated in peripheral laboratories with minimal infrastructure and is therefore considered to be the first WHO-recommended molecular near-point-of-care test for TB and RIF resistance.^2^

The early treatment of TB is dependent on early diagnosis, and the turnaround time (TAT) for diagnosis is a major factor. The WHO approved Truenat, especially for peripheral laboratories facing power challenges and remote areas with limited infrastructure. This near-point-of-care test is expected to reduce the TAT of TB diagnosis in peripheral laboratories.^3,4^

A major factor contributing to low TB case detection in Nigeria is limited access to rapid molecular testing. In 2021, although the proportion of presumptives who underwent the Xpert (Cepheid, Sunnyvale, CA, USA) test increased to 79%,^1^ a considerable number of presumptives (21%) still underwent acid-fast bacilli microscopy as the initial diagnostic test. The major reasons for using microscopy include but are not limited to prolonged Xpert machine downtime due to modular, infrastructural, and power challenges, and a suboptimal specimen referral system.

Given the challenges of running Xpert tests and the need to promote quick access to diagnosis for presumed TB patients, Molbio's Truenat MTB Plus and MTB-RIF Dx testing platform recommended by the WHO in 2020 was deployed to peripheral facilities within Nigeria. One of the key advantages of this technology compared with other available WHO-recommended molecular diagnostic (mWRD) platforms is that it can be placed closer to patients at the lowest level of health facilities. This approach can reduce the long waiting time between sample collection, referral to a testing laboratory, and the eventual release of results, thereby reducing patient management delay.

To address low rates of TB case detection and support Nigeria in the fight against TB, the United States Agency for International Development (USAID), through the Stop TB Partnership, introduced the New Tools Project, which donated 38 Truenat platforms to Nigeria. The machines were installed across 14 states in the country, and implementation began in December 2021. The current study aimed to evaluate the impact of the deployment of Truenat testing on TB diagnosis at peripheral facilities.

## METHODOLOGY

### Study setting

**FIGURE 1.**
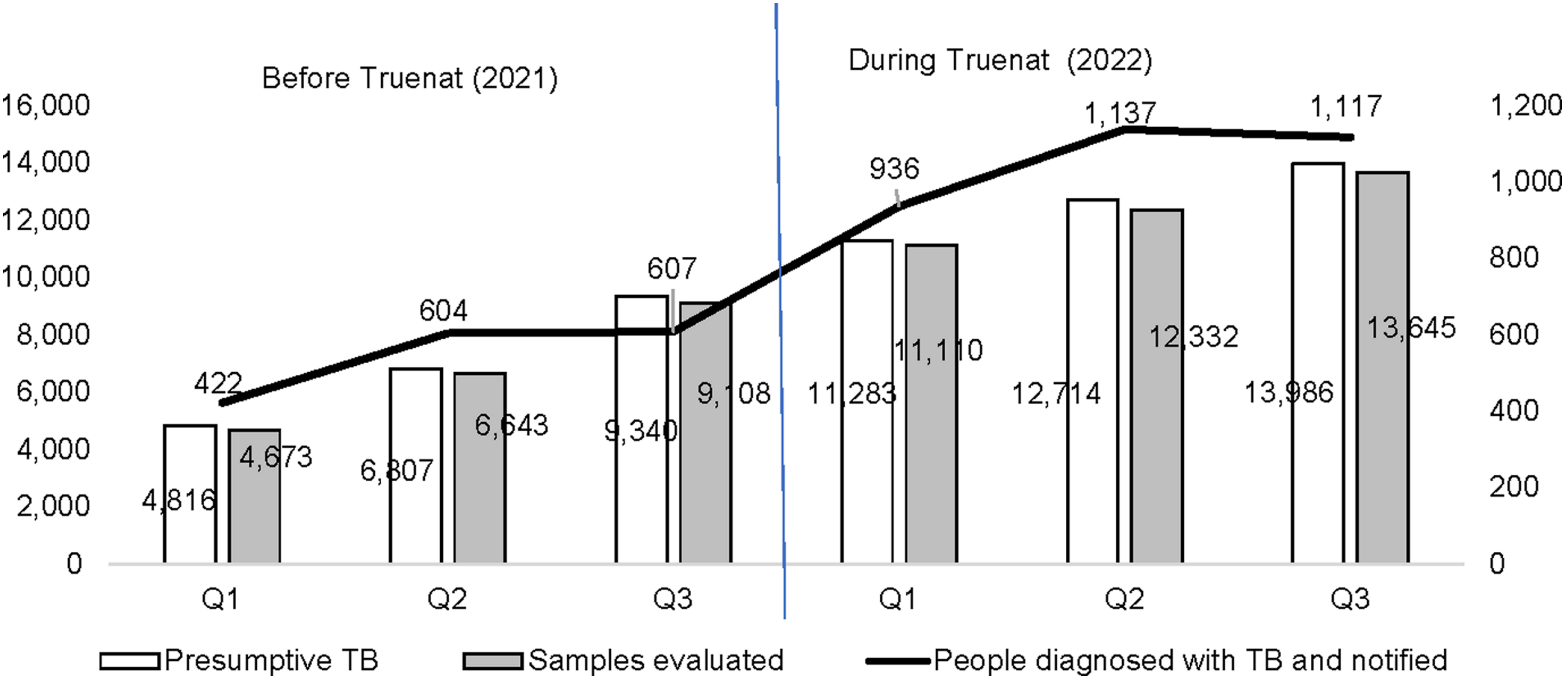
Comparison of presumptive TB cases, evaluation and number of TB cases notified before and after the introduction of Truenat testing.

This study was conducted in 14 USAID-supported TB LON countries. The States where Truenat machines were deployed are shown in Figure 1 below and include Kano, Katsina, Kaduna, Bauchi, Nasarawa, and Taraba in the north, while Akwa-Ibom, Anambra, Cross River, Delta, Rivers, Lagos, Osun, and Oyo States are located in the southern part of the country. The 38 Truenat machines are located in peripheral laboratories across the USAID-supported LON 1, 2, and 3 States. Lagos, Osun, and Oyo are part of the USAID-supported TB-LON 3 project implemented by the Institute of Human Virology Nigeria (IHVN), with the remaining 11 states under USAID-supported LON Regions 1 and 2, implemented by the KNCV Tuberculosis Foundation, Lagos, Nigeria.

### Study design

This was a retrospective cross-sectional multicentre study that included 34 of the 38 Truenat implementing facilities in Nigeria. Of the 38 Truenat sites in Nigeria, 34 are located in facilities that offer DOTS services, while four of the sites are standalone testing laboratories without DOTS services. Information about TAT to TB diagnosis and time to commencement of TB treatment were obtained. The trends in TB case notification before and after the roll-out of the point-of-care and near-point-of-care Truenat assays for the diagnosis of pulmonary TB were compared. The study also involved quantitative analysis of retrospective secondary data on TB case-finding and selected parameters before and after the Truenat roll-out.

### Study population

The study population consisted of clients who accessed Truenat tests within the study period, as recorded in the laboratory registers of the study sites, as well as patients with records in the presumptive and treatment registers of the study facilities.

### Sample size determination

Total sampling was used in the study. All participants with clearly documented information within the nine-month study period (Q1–Q3 of 2022, i.e., January–September 2022) were included in the study. The derived information was used to determine the TAT for TB diagnosis and treatment. The findings for TB case notifications for the same period (Q1–Q3 of 2022) were compared with the corresponding quarters (Q1–Q3 of 2021) before Truenat's introduction.

### Study procedures

Research assistants were recruited from Truenat implementation states and trained in data collection using data abstraction tools. Secondary data from the presumptive, treatment and laboratory registers at Truenat sites were also collected by the research assistants for analysis using the data abstraction tool. The collected data included data from the 9-month period before (Q1–Q3, 2021) and post-rollout (Q1–Q3, 2022) Truenat at implementation sites across the country.

### Exclusion criterion

All participants with incomplete data were excluded from the study.

### Data collection

Study tools included the laboratory registers of the study sites, presumptive TB registers, and TB treatment registers of the study sites.

### Outcome measures

The primary outcome measures included the TAT for the bacteriological diagnosis of TB at the Truenat sites and the time to treatment initiation for bacteriologically diagnosed TB cases at the Truenat sites determined from the facility's presumptive and treatment registers, respectively. The primary outcome measures are defined as follows:

- Turnaround time for bacteriological diagnosis: The number of days from the date of sputum specimen collection from the presumptive TB patient to the date the laboratory result was received as recorded in the clinic presumptive TB register.
- Time to treatment initiation: The number of days from the date of receipt of the TB test result to the date the diagnosis of the patient was initiated on TB treatment, as recorded in the TB treatment register.

The study outcome measures also included the proportion of newly diagnosed patients with TB at Truenat sites who initially underwent rapid molecular testing as an initial test.

### Data management

Data were directly collected from the laboratory and TB DOTS sites and entered into an Excel spreadsheet by the data collectors. The data collected at each state level were reviewed and cleaned by the Data Officer before being shared with the statistician on the central study team for review and further validation before analysis. Data were analysed using International Business Machines, Statistical Product for Service Solutions (IBM-SPSS) statistical software v25 (IBM, Armonk, NY, USA). Summary statistics were generated and presented in frequency tables and charts.

### Ethical consideration

Ethical approval for this study was obtained from the National Health Research and Ethical Committee (NHREC) before the commencement of the study. The approval sought included waiver of consent given that retrospective de-identified data were used in the study. No personal identifiers were extracted to ensure confidentiality, and the participants' unique identification numbers were used in the data collection tools. However, the data collectors signed a non-disclosure agreement.

## RESULTS

Of the 2,335 entries received, 1,636 (70.1%) had complete information for determining the time to diagnosis, whereas of the 1,070 entries, 45.8% had complete information for determining the time to treatment initiation. Almost all RIF-resistant cases were referred from facilities where they were diagnosed and were therefore not included in this study.

Tables 1A and 1B show turnaround times from diagnosis to initiation of TB treatment. The median time to diagnosis of TB was zero (<24 h) (IQR 0–1 day). Of the total sample of 1,636 cases, the majority of 57.9% were diagnosed in <24 h, whereas 20.9% were diagnosed within 24 h. Moreover, 7.9% of the samples were diagnosed within 48 h, whereas 5.2% were diagnosed within 72 h.

The median time to commencement of treatment for 1,070 newly diagnosed patients with TB was 1.5 days (IQR 0–3). The time from sputum collection to initiation of TB treatment was <24 h for 28.1% of patients and within 24 h for 21.9% of newly diagnosed patients with TB. It took 48 h to initiate treatment for 14.7% of the patients, and 11.4% had their treatment initiated within 72 h. Moreover, 17 (1.6%) patients commenced treatment for TB after two weeks.

**TABLE 1A).** Turnaround time for diagnosis and commencement of treatment.

Time interval	Median
	[IQR]
Time to diagnosis, days (*n* = 1,636)	0 [0–1]
Time to commencement of treatment, days (*n* = 1,070)	1.5 [0–3]

**Table:** **B).** Proportion of TB cases diagnosed and commenced treatment on specific days.

Time interval	<24 h	24 h	48 h	72 h	>72 h
	*n* (%)	*n* (%)	*n* (%)	*n* (%)	*n* (%)
Time to diagnosis, days (*n* = 1,636)	947 (57.9)	342 (20.9)	129 (7.9)	85 (5.2)	132 (8.1)
Time to commencement of treatment, days (*n* = 1,070)	301 (28.1)	234 (21.9)	157 (14.7)	122 (11.4)	256 (23.9)

**FIGURE 2.**
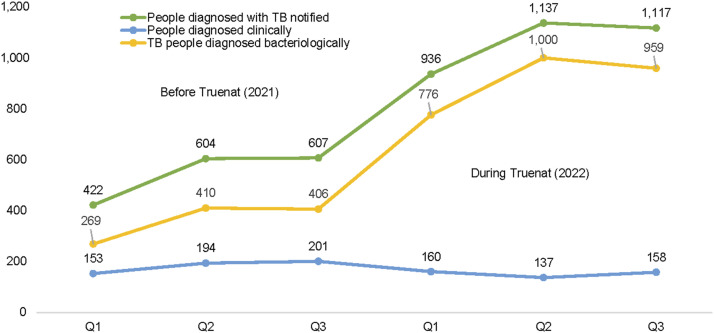
Line graph showing comparisons between case notification and mode of diagnosis (bacteriological and clinical) before and after the introduction of Truenat testing.

**FIGURE 3.**
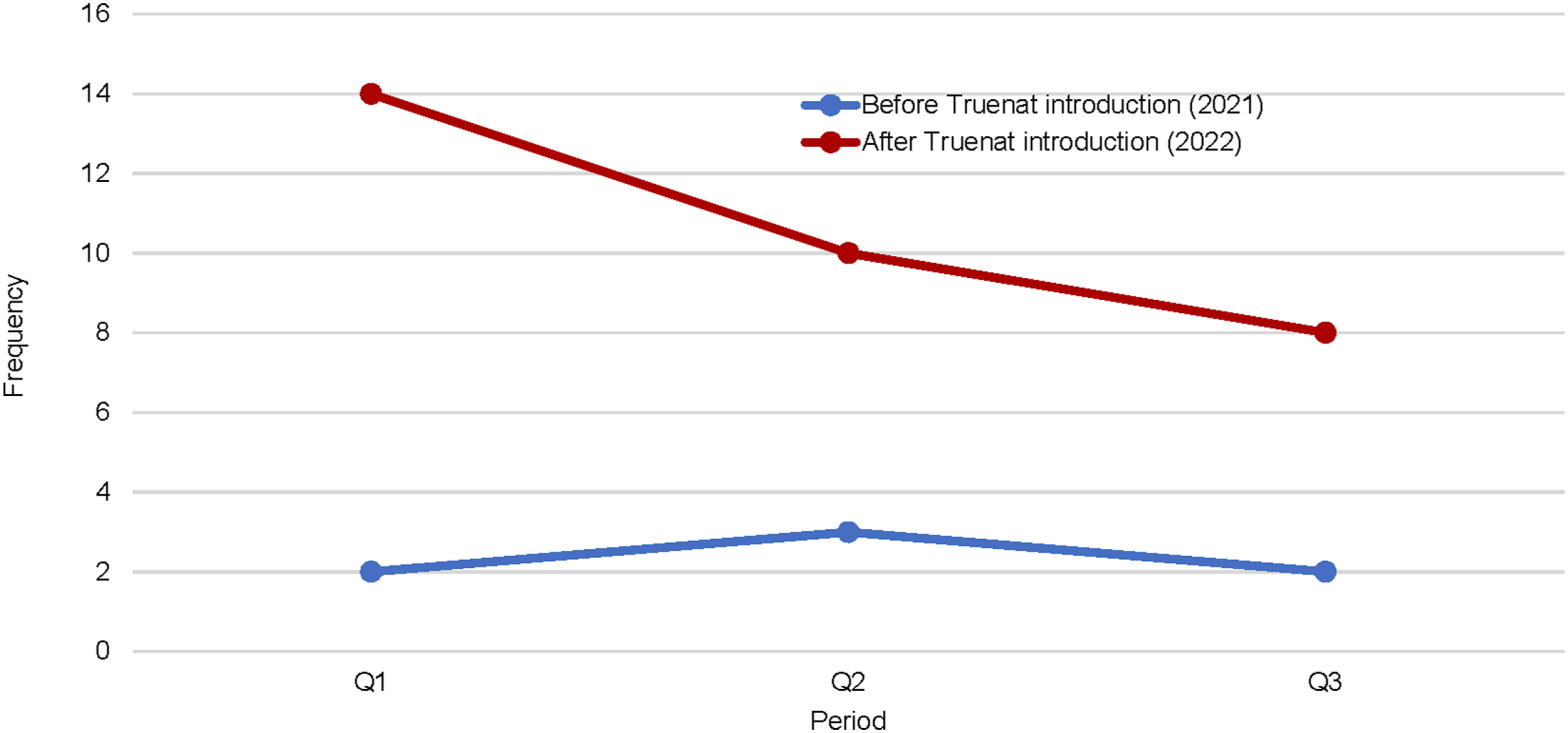
Line graph showing the number of RR-TB cases detected before and after the introduction of Truenat. RR-TB = rifampicin-resistant TB.

This study has demonstrated the remarkable impact of Truenat testing on the TB care cascade; we compared three similar quarters before (Q1, Q2, and Q3, 2021) and after (Q1, Q2 and Q3, 2022) installation of Truenat platforms in the supported facilities (see Table 2). We observed an increase across the TB cascade between pre- and post-installation data as follows: 37,983 (81%↑) in the number of individuals with presumed TB identified, 37,087 (82%↑) evaluated, 3,190 (95%↑) TB cases notified and 32 (357%↑) in RR-TB case notified. Compared with 20,963 presumptive, 20,424 were assessed, 1,633 were notified, and 7 RR-TB cases were notified before the installation of Truenat in the facilities (see Figures 1 and 2). Worthy of mention is the significant reduction in the number of clinically diagnosed TB cases after the introduction of Truenat from 548 (17%↓) to 455 TB cases after the installation of Truenat (see Figure 3).

**TABLE 2.** Comparison of TB case notifications before and after the introduction of Truenat.*

	Before Truenat (2021)				During Truenat implementation (2022)			
Variable	Q1	Q2	Q3	Total	Q1	Q2	Q3	Total
Presumptive TB, *n*	4,816	6,807	9,340	20,963	11,283	12,714	13,986	37,983
Samples evaluated, *n*	4,673	6,643	9,108	20,424	11,110	12,332	13,645	37,087
Newly diagnosed people with TB notified, *n* (%)	422 (9)	604 (9)	607 (7)	1,633 (8)	936 (8)	1,137 (9)	1,117 (8)	3,190 (9)
Newly developed TB diagnosed clinically, *n*	153	194	201	548	160	137	158	455
Newly developed TB diagnosed bacteriologically, *n*	269	410	406	1,085	776	1,000	959	2,735
Bacteriologically confirmed TB, %	64	68	67	66	83	88	86	86
Newly diagnosed TB patients using Truenat, *n*	0	0	0	0	698	917	863	2,478
Newly diagnosed TB using mWRD, %	0	0	0	0	89.9	91.7	90.0	90.6
RR-TB diagnosed	2	3	2	7	14	10	8	32

* Comparison of the 34 health facilities implementing Truenat.

mWRD = WHO-recommended molecular diagnostic; RR-TB = rifampicin-resistant TB.

**FIGURE 4.**
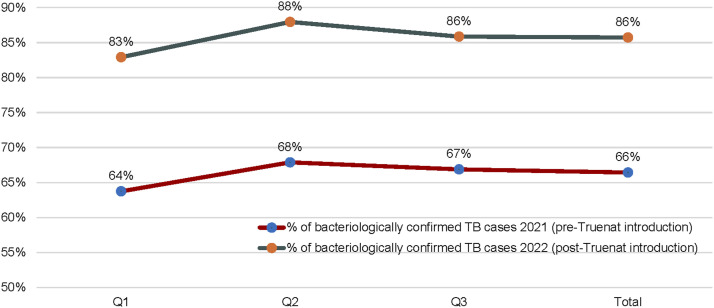
Proportion of bacteriologically confirmed TB cases before and after the introduction of Truenat.

## DISCUSSION

This study showed that the number of individuals with presumed TB identified, the number of samples tested, and the TB yield all increased with the introduction of Truenat to peripheral facilities compared with the figures from the corresponding quarters of the previous year before the introduction of Truenat. Moreover, close to 81% of the results were issued within 24 h in health facilities using Truenat, with an average time to diagnosis of 1.0 ± 2.0 days and an average time to initiation of treatment of ±1.5 days. The findings are better than the baseline results from another Nigeria study that assessed the effect of a pilot intervention (Finding, Actively, Separating and Actively, FAST) on time to diagnosis and treatment in tertiary and referral health facilities using GeneXpert. In the FAST study, the intervention resulted in a reduction in the time to diagnosis from a baseline of 2.9 days to 1.9 days, whereas the time to initiation of TB treatment was reduced from 3.9 days to 1.1 days.^5^

The time to diagnosis and treatment initiation are crucial determinants of health system delays, which form part of the total delay experienced by patients with TB experiences during patient pathway analysis. Early diagnosis and treatment are foundational to TB control efforts, as early treatment reduces mortality and ensures a break in the cycle of transmission.^6^ In a retrospective study in Benue State, Nigeria, the majority of patients diagnosed with TB commenced treatment after 3 weeks of diagnosis. The same study found a higher treatment failure and death rate among patients who commenced their treatment late.^7^ Another study in Nigeria attributed early deaths in a retrospective cohort of TB patients to delay in diagnosis and treatment for TB amidst other factors.^8^ From the results of our study, Truenat in peripheral sites decreased the time to diagnosis and commencement of treatment, thereby reducing health system delays. However, 1.6% of the participants started treatment after two weeks. The reasons for commencing treatment late for TB include a lack of short message service (SMS) notifications, individuals not returning to check their results, and an inadequate number of community health workers who could have traced the individuals to bring them back to the health facility for treatment. Electronic transmission of test results has been piloted in some health facilities in Nigeria, and this approach has helped to reduce the time to initiate treatment for TB. It is expected that when this electronic transmission of results becomes the norm, the time to initiate treatment for TB will be greatly reduced. This approach is expected to reduce community TB transmission, pre-treatment loss to follow-up, and TB-associated mortality and improve the linkage to care and quality of TB patient service delivery.^6,9^

In the first three quarters of 2021, before the introduction of Truenat, 5.3% of the 20,424 presumptives tested were bacteriologically diagnosed with TB, whereas after the introduction of Truenat, the yield increased to 7.4% of the 37,087 identified presumptives. Our study demonstrated a greater impact of a 357% increase in the number of RIF-resistant cases diagnosed at the study sites. The fact that 91% of all bacteriologically diagnosed patients in Truenat facilities tested with Truenat during the intervention period reflects increased access to molecular diagnosis. Before the introduction of Truenat, these facilities relied on sample logistic systems to transport their presumptive sputum specimens to the nearest GeneXpert sites. At other times, samples were analysed using AFB microscopy. The study results demonstrate the strong impact of Truenat in increasing access to molecular diagnosis for TB in challenging health systems, similar to reports from a previous study in India.^2^ Our study results also align with one of the WHO strategies of increasing access to molecular diagnostics to help identify missing TB cases.^10,11^

Nigeria is among the 10 countries that collectively account for 75% of the global missing TB cases and the 10 countries that account for approximately 70% of the missing DRTB cases.^1^ This is against a background of poor access to molecular WHO-recommended diagnostic tests and the resulting low DR-TB notification rates for Nigeria, which are some of the major challenges facing the Nigeria TB Program.^12^ The National TB Programme strongly prioritises the identification of missing TB cases in its National Strategic Plan and boldly proposes the scale-up of molecular WHO-recommended diagnostics, including Truenat, as part of its key intervention strategies.^13^ The WHO believes that improving DR-TB testing, diagnosis, and access to treatment in countries like Nigeria, which account for the global gap in missing DR-TB cases, will increase the treatment coverage of DR-TB globally.^1^ Suffice it to say that from the results of this study, the deployment of Truenat increased the access to molecular diagnosis of TB, increased the proportion of bacteriologically diagnosed TB cases, and increased the number of DR-TB cases diagnosed and thus a recognised approach to finding the missing TB cases in Nigeria.

### Study limitations

Some entries at the Truenat sites (29.1%) did not have complete information, including dates for determining turnaround times. These issues, especially those related to incomplete information, may have affected the results of this study. Given these observations, it is recommended that DOTS providers be trained on the need for completeness of data entries, especially metrics for determining quality and timeliness of care.

## CONCLUSIONS

The deployment of Truenat increased the detection of TB and DR-TB cases in peripheral facilities in Nigeria. It also decreased the time to diagnosis and time to initiation of TB treatment. These findings have positive implications in the fight against TB, and Truenat is relevant for identifying missing TB cases in Nigeria.
